# Discovering and validating between-subject variations in plasma lipids in healthy subjects

**DOI:** 10.1038/srep19139

**Published:** 2016-01-08

**Authors:** Husna Begum, Bowen Li, Guanghou Shui, Amaury Cazenave-Gassiot, Richie Soong, Rick Twee-Hee Ong, Peter Little, Yik-Ying Teo, Markus R. Wenk

**Affiliations:** 1 NUS Graduate School for Integrative Science and Engineering, National University of Singapore, Singapore; 2 Department of Biochemistry, Yong Loo Lin School of Medicine, National University of Singapore, Singapore; 3 Life Sciences Institute, National University of Singapore, Singapore; 4 Saw Swee Hock School of Public Health, National University of Singapore, Singapore; 5 State Key Laboratory of Molecular Developmental Biology, Institute of Genetics and Developmental Biology, Chinese Academy of Sciences, Beijing, China; 6 Cancer Science Institute of Singapore, National University of Singapore, Singapore; 7 Genome Institute of Singapore, Agency for Science, Technology and Research, Singapore; 8 Department of Statistics and Applied Probability, National University of Singapore, Singapore; 9 Department of Biological Sciences, National University of Singapore, Singapore

## Abstract

Lipid levels are commonly used in clinical settings as disease biomarkers, and the advent of mass spectrometry-based (MS) lipidomics heralds the possibility of identifying additional lipids that can inform disease predispositions. However, the degree of natural variation for many lipids remains poorly understood, thus confounding downstream investigations on whether a specific intervention is driving observed lipid fluctuations. Here, we performed targeted mass spectrometry with multiple reaction monitoring across a comprehensive spectrum of 192 plasma lipids on eight subjects across three time-points separated by six hours and two standardized meals. A validation study to confirm the initial discoveries was performed in a further set of nine subjects, subject to the identical study design. Technical variation of the MS was assessed using duplicate measurements in the validation study, while biological variation was measured for lipid species with coefficients of variation <20%. We observed that eight lipid species from the phosphatidylethanolamine and phosphatidylcholine lipid classes were discovered and validated to vary consistently across the three time-points, where the within-subject variance can be up to 1.3-fold higher than between-subject variance. These findings highlight the importance of understanding the range of biological variation in plasma lipids as a precursor to their use in clinical biochemistry.

Lipids are involved in all systems of human physiology, and their abundance is governed by the interplay between genetics, diet and environment. Indeed, lipid metabolism forms the central basis for structure and energy homeostasis, as well as for physiological communication such as in inflammation. In blood, the upper and lower reference levels for lipid content are well described for only a few lipids, such as that of blood cholesterol and triacylglycerol. Although both lipids are clinically used as risk markers for disease onset (e.g., cardiovascular disease), the natural variation of these lipids within an individual is poorly documented or understood, and reference levels in the population have almost entirely been determined on the basis of lipids in blood obtained after a period of fasting. This is similarly true for the large number of blood lipids that can now be quantified with modern mass spectrometry technologies in the study of lipids.

Metabolites tend to vary substantially between individuals and on a day-to-day basis^1^, and such fluctuations complicate comparative studies. Often, the degree of natural variation of a metabolite/lipid in an individual and a relevant reference population is not known. A recent study of twenty healthy male subjects, all of whom are within a relatively narrow age range and assessed in a controlled laboratory environment, showed extensive diversity in the circadian regulation of plasma lipids and provided clear evidence for different circadian metabolic phenotypes^2^.

Clinical biochemistry has clearly demonstrated that the concentration ranges of a number of blood metabolites depend on the time of day (cortisol), gender (hormones, creatinine), and ethnicity (free fatty acids)^3^. Lipid reference values are emerging for specific lipid metabolites as part of clinical lipoprotein fractions (such as HDL, LDL) and, more recently, for total blood/plasma concentrations. Indeed, the effects of age^4^, gender and ethnicity^5^ on plasma lipid concentrations have been determined by mass spectrometry in a healthy subject cohort representative of the US population. Together with other examples, these studies showed altered levels of cholesterol^6^, glycerophospholipids (such as glycerophosphorylcholine), and free fatty acids (linoleic acid) among groups of healthy subjects as a function of age, gender and ethnicity^7^.

Worryingly, the majority of existing literature around mass spectrometry lipidomics has overlooked the importance of measurement reproducibility and instrument validation. This is increasingly a concern in lipidomics research as reports are surfacing about how biological findings are confounded by instrumental errors, leading to a failure to reproduce previously observed experimental findings^8^,^9^,^10^.

In this study, we aim to evaluate the extent of within-subject variation in levels of plasma lipids, relative to the variation seen between subjects. Plasma lipid levels were measured in a pilot panel of eight healthy subjects across three time-points of the same day to discover the extent that blood lipids vary between fasting and post-meal intakes, and lipid species which were identified to vary significantly were evaluated in a further validation panel of nine healthy subjects. The subjects in the validation panel were assayed twice in order to ensure that any discoveries were genuinely due to biological changes and not artefacts due to instrumental or technical variation.

## Results

**Figure 1:**
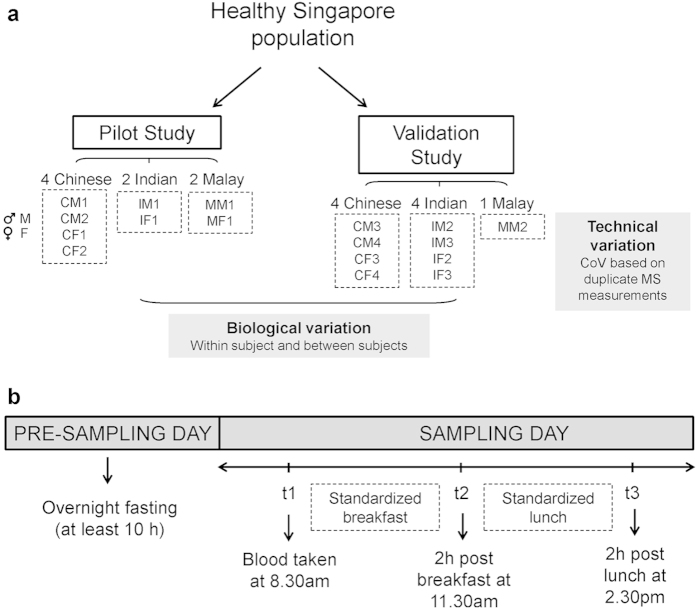
General outline of the pilot and validation studies. (**a**) Study population distribution. Two study cohorts were used for the pilot and validation studies. The validation study was performed on a different set of subjects (duplicate measurements) to confirm the pilot study findings and to determine technical variability. Biological variation was assessed in both studies (data in text). Ethnic coding: C = Chinese, I = Indian, M = Malay. Gender coding M = Male and F = Female. (**b**) Schematic representation of study design and blood sampling. All samples were taken within the same day for each study across the three time-points (t1 to t3).

The pilot cohort of eight subjects contained an equal distribution of males to females, and consisted of participants from the three major ethnic groups in Singapore, comprising Southern Han Chinese, Southeast Asian Malays and South Asian Indians (Fig. 1A). Owing to the small sample sizes, there were no observable differences in lipid profiles as a result of gender or ethnic differences, and subsequent analyses did not consider gender or ethnicity (Supplementary Figure 1). Lipid profiles across 128 lipid species were measured from blood plasma samples taken at three time points: (i) after a period of overnight fasting (t1; Fig. 1B); (ii) two hours after a standardized breakfast (t2); and (iii) two hours after a standardized lunch (t3).

**Figure 2:**
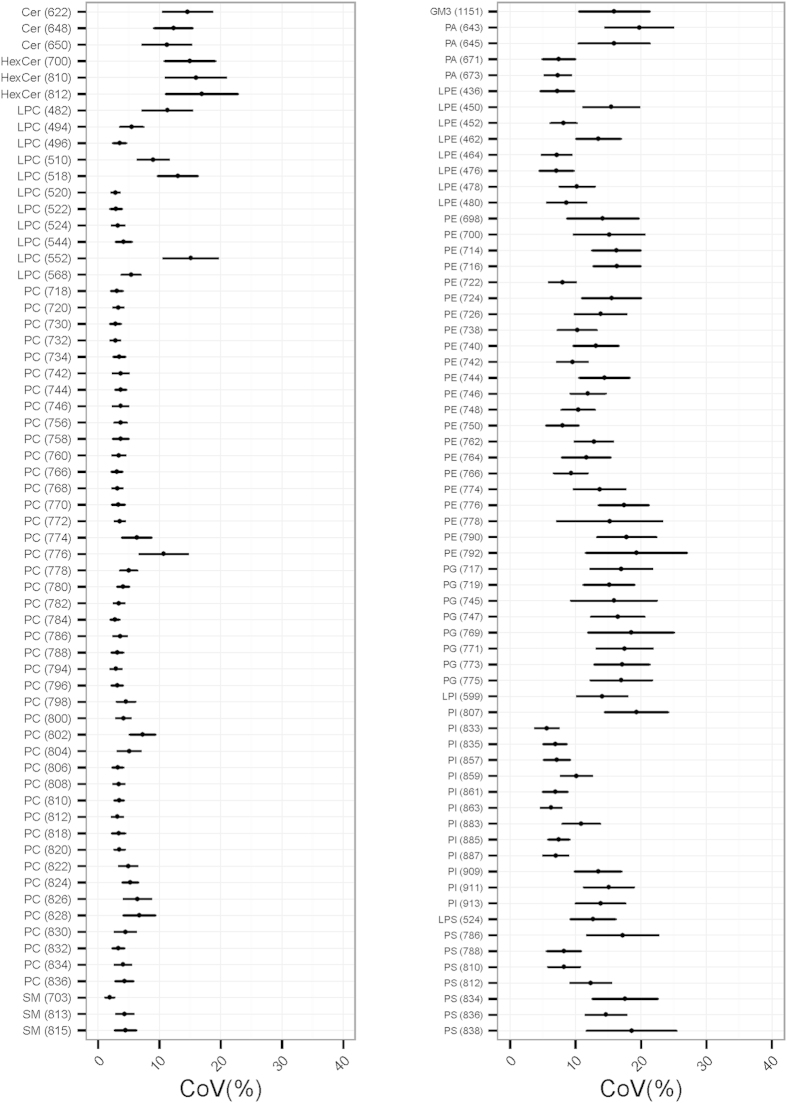
Extent of technical variation. Coefficient of variation (CoV) is shown across 63 (positive ionization, left panel) and 65 (negative ionization, right panel) lipid species measured via mass spectrometry (MS). CoV is represented by mean and 95% confidence interval (2 technical replicates of 26 samples) of each lipid species in the validation batch. Lipid species are annotated by their lipid class followed by their parent ion mass measured by tandem MS; e.g., PC (834) = phosphatidylcholine species measured at 834 precursor ion *m/z*.

Our analyses for time-dependent variation specifically focused on whether there was any evidence for monotonic increases or decreases in the measurements of each lipid species across the three time points. We observed nine lipid species with preliminary evidence of time-dependent variation, where the levels of all nine lipids exhibited an increasing trend with time (Table 1). To evaluate whether this observation was reproducible, a validation panel of another nine healthy subjects was recruited with exactly the same dietary and multiple time-point sampling protocol (see Fig. 1). Each of the plasma samples taken from the validation panel was also assayed twice to evaluate the technical reproducibility of the MS lipid measurements, where we observed that none of the nine lipids identified in the discovery panel exhibited a coefficient of variation (CoV) > 20% (Fig. 2), which is the benchmark for analytical reproducibility in lipid measurements^11^.

**Figure 3:**
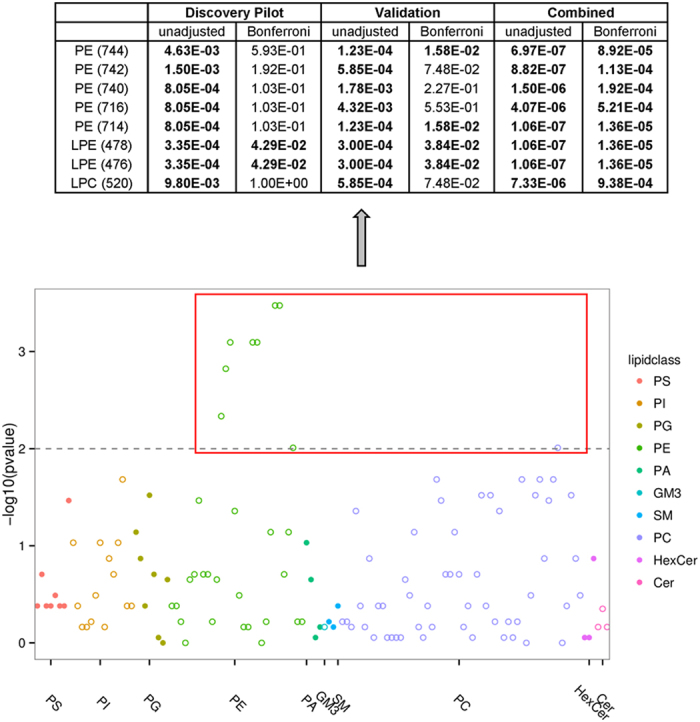
Global profile showing –log10 p-values of 128 measured lipid species across three timepoints t1 to t3 (unadjusted). Horizontal line indicates Bonferroni correction of –log10 (p-value = 0.01). Eight lipid species are significantly altered across t1 to t3 after Bonferroni correction across both discovery pilot and validation studies. The identities of these eight species are represented in the table above before and after Bonferroni adjustment across both independent and combined studies.

**Figure 4:**
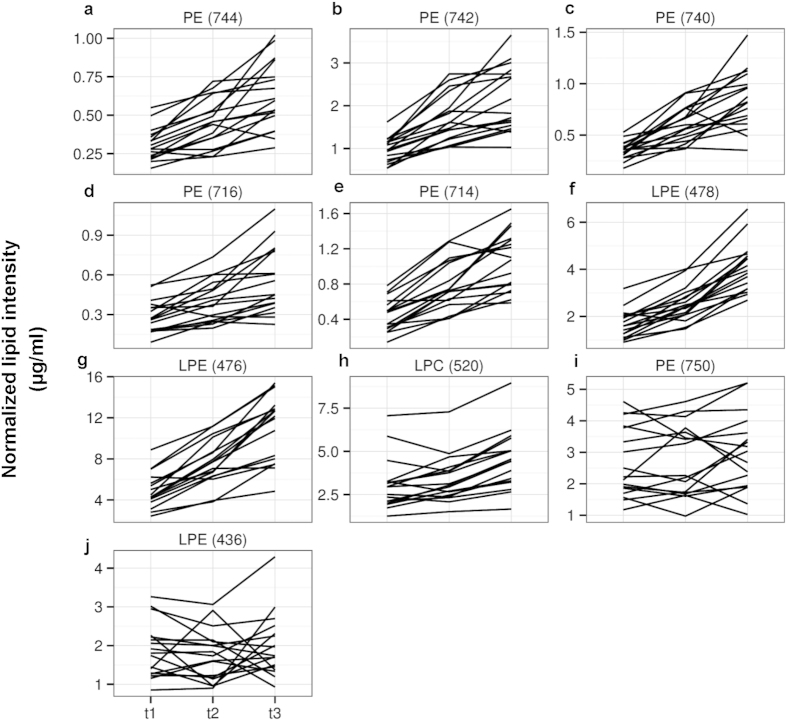
Representative lipid species significantly altered across the three time-points (t1 to t3). Panels (**a**–**h**) show representative lipid species significantly altered across t1 to t3, in contrast to lipid species with no significant differences across the time-points shown on panels (**i**–**j**). Technical Coefficient of variation (CoV) for all these species are less than 15% (Fig. 2). Y axis indicate normalized lipid intensity (μg/ml) across all 17 subjects (discovery pilot and validation studies combined). t1 = baseline readings; t2 = post-breakfast readings; t3 = post-lunch readings. Meals were standardized for each subject.

We observed that eight of the nine discovered lipids presented concurring evidence of consistent time-dependent variation in the validation panel, of which eight lipid species presented combined evidence more significant than the naïve Bonferroni-adjusted threshold of P < 3.9 × 10^−4^ (Table 1, Fig. 3), such that these eight lipid species consistently increase with time (Fig. 4). In particular, we noticed that these eight lipid species each contained at least one 18-carbon fatty acyl chain and were predominantly from the phosphatidylethanolamine (PE) class, at mass to charge ratios (*m/z*) of 476, 478, 716, 714, 740, 742, and 744, but also included a member from the phosphatidylcholine (PC) class (*m/z* of 520).

**Figure 5:**
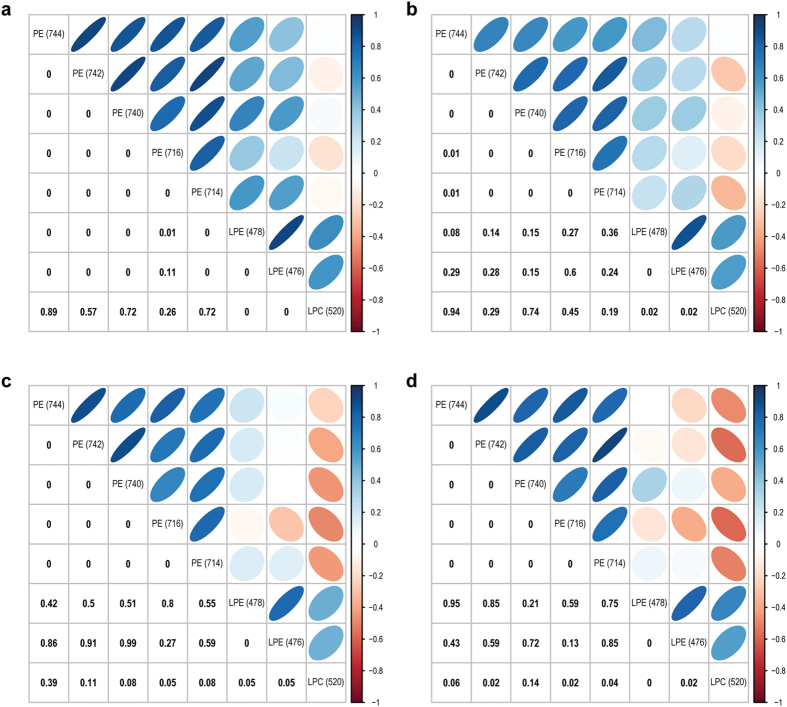
Correlation analysis showing p-values and correlation coefficients across the eight lipid species significantly altered across timepoints t1 to t3. Correlation p-values are shown numerically (bottom left) and correlation coefficients are represented by colour and shape (top right). Darker blue indicates stronger positive correlation and darker red indicates stronger negative correlation. Thinner ellipse indicates stronger correlation, and rounder oval indicates weaker correlation. (**a**) All timepoints (t1 to t3) (**b**) t1 (**c**) t2 (**d**) t3.

While the eight lipid species were monotonically increasing with time, a correlation analysis of the lipid measurements at each of the three time-points revealed that lipid species from the same classes tend to be highly correlated (Fig. 5). For example, the measurements of two PE species (*m/z* 714, 744) across the 17 subjects from the discovery and validation panels were correlated across all three time points, (t1: correlation r = 0.58, P = 1.4 × 10^−2^; t2: r = 0.74, P = 6.8 × 10^−4^; t3: r = 0.80, P = 1.2 × 10^−4^). This correlation however was not observed across lipid classes, even between lipid species from the PE and lyso-PE classes.

## Discussion

We have evaluated how plasma lipids vary across three time points in two panels of healthy subjects, and identified eight lipid species which reproducibly exhibited evidence of increasing consistently with time. These eight lipid species were also robust to any human or instrument variation with low levels of CoVs when comparing between the measurements from technical duplicates in the validation arm. The sampling protocol was deliberately established within the same day and timeframe to account for any possible metabolic variations as a result of circadian rhythms.

Lipid species that varied significantly with time (see Table S1 for raw data and processed data) exhibited a time-dependent increasing trend in all subjects, irrespective of gender and ethnicity, and these changes were consistent with meal intake. Although the observed changes could be due to differences in lifestyle factors (e.g., dietary habits), a single day of dietary standardization might be sufficient to normalize the data for determining differences, as the subjects remained stable in their metabolic space after 24 hours^12^. Thus, it is possible that significant changes in lipid levels observed in this study could be metabolically important. Indeed, the lipid species identified to be significantly different in their temporal concentrations are commonly found in circulating lipoproteins^13^. Dietary lipids have been shown to be incorporated into liver lipids^14^ and are subsequently expected to more widely affect blood phospholipids^15^. Longitudinal study designs, such as the one used here, will therefore be pertinent approaches to follow lipid distributions and thus metabolism.

Six plasmalogen PE and PC species (though not statistically significant) were identified to be associated with time and this result is consistent with a previous study^16^. In particular, PE (728) or (36:1p and/or 36:2e) and PE (812) or (42:1p and/or 42:2e) were found to be increasing across the three time-points in our study, whereas PC (746) or (34:0p and/or 34:1e), PC (768) or (36:3p and/or 36:4e), PC (766) or (36:4p and/or 36:5e), PC (824) or (40:3p and/or 40:4e) species were found to be decreasing with time. These differences could be due to the dynamic nature of plasmalogen lipids, which have been reported to have a high turnover rate, thus resulting in dynamic inter-individual variations across time^17^. In this study, a MS approach was used which has an added benefit of yielding information on the likely fatty acid compositions present in these lipid species, unlike non MS approaches used in some of the previous studies.

Previous studies have also investigated the effect of physiological challenges on the human metabolome, including exercise and cold stress, and have revealed that the human metabolome is subjected to dynamic variations^18^, including day-to-day variations^19^,^20^. Here, we show that, even within a controlled dietary and sampling setting, there is an important need to be consistent in collection times, as significant differences in lipid levels occur at different times of the day and further affected by meal intake within the same subject.

Here, we have found that the within subject variation appears to be smaller than between subject variation indicating that biological variation between individuals are still larger than dynamic within subject variability (Supplementary Figure 1). The within-subject variation in lipid levels observed in this study could be due, at least in part, to the effects of the circadian clock. In the blood plasma, lipids represent more than 75% of all rhythmic compounds^19^, and many lines of evidence are in support of the concept that time of day-dependent rhythms in phospholipid metabolism are driven by autonomous circadian clocks^21^. For example, changes in external factors, such as food availability (fasting and feeding), drive daily rhythms in lipid metabolism^22^.

We will like to emphasize that the eight lipid species have been identified through a stringent statistical process which properly addressed the issue of multiple testing. This means that the sample size attained the necessary specificity to identify the large effects observed at these eight lipid species. However, we expect the stringency comes at the cost of reduced sensitivity. Taken together, this means we are confident that the discovery of the eight lipid species is unlikely to be artefactual, but we may have inevitably failed to identify other lipid species that displayed weaker evidence of consistent time-dependent fluctuations due to the smaller sample size.

Biological variations in longitudinal measurements and within-subjects measurements are important considerations, primarily due to heritability and environmental factors that result in different metabolite profiles^8^,^23^. It is imperative that technical variation is established prior to discerning biological variation so that more robust lipid biomarkers can be ascertained. In this study, these eight lipid species may not be useful candidates to be biomarkers of various pathological conditions due to their large within subject variations.

We have shown that using well characterized laboratory procedures with robustly defined ranges of technical variation (CoV > 20%), we are able to reliably measure lipid levels without confounding significant biologicals findings. Establishing lipid variations within subject is important as it further opens the field to study biological variation across different ethnic groups in multi-ethnic populations such as Singapore.

## Methods

### Subjects and study design

All healthy human plasma samples were collected in accordance to ethical guidelines and protocols. This study was approved by the National Health Group Institutional Review Board (IRB 10-434), Singapore. All subjects who were approached and who agreed to participate in this study were required to provide written consent. Informed consent was obtained from all subjects. Subjects could withdraw, refuse to participate or discontinue at any point during the study without further prejudice. All subjects were of Singaporean nationality, with the cohort consisting of four Chinese, two Malay and two Indian subjects for the pilot study, and four Chinese, four Indian and one Malay subjects for validation (Fig. 1A). The ethnicity of each subject was confirmed verbally by obtaining information of their grandparents’ and parents’ ethnicities. Subjects did not have any existing major health conditions, such as diabetes and cardiovascular disease, although no detailed clinical assessments were made. Female subjects were ensured to be not pregnant at the time of sampling. Samples were collected by trained phlebotomists. All subjects had fasted overnight and blood was taken after a minimum of 10 hours of fasting (only the consumption of water was allowed during the fasting period). A standardized breakfast (same meal) was given to all participants within 30 min of the first blood draw. Blood was drawn again two hours after breakfast was consumed. Subjects were then given a standardized lunch (same meal) and the last time-point for blood collection was performed two hours after lunch was consumed (Fig. 1B).

### Blood sample collection and plasma processing

For plasma samples, 10 ml of whole blood was collected by venipuncture in BD Vacutainer® plastic plasma tubes with K2 EDTA (#366643, lavender closure) as an anticoagulant. Tubes were inverted 3–5 times to allow adequate mixing of the blood with the anticoagulant. The blood was spun at 2200 g for 15 min using a pre-cooled, 4 °C swing-out bucket rotor centrifuge (Allegra 6R Centrifuge, Beckman Coulter, Fullerton, CA). The top plasma layer was then removed by carefully pipetting out the supernatant and placed in the cryovials (Practical Mediscience, Singapore); ~500 μl of plasma near the plasma–blood interface was avoided so as to reduce contamination by platelets. All plasma samples were mixed, frozen at −20 °C, and then frozen to −80 °C within 45 min of blood collection. Subsequently, plasma samples were divided into 100-μl aliquots and stored at −80 °C for lipid extraction and analysis. All samples underwent the same number of freeze–thaw cycles to ensure consistency in the sample processing. The effect of freeze-thaw cycles was not studied as previous publications have already showed that there are minimal changes to metabolites when subjected up to three freeze thaw cycles^24^.

### Lipid extraction

Pre-chilled solvents were used throughout lipid extraction. Lipids were double extracted according to a modified Bligh and Dyers extraction method^25^. Thawed plasma (100 μl) was used for lipid extraction, as previously described^26^. Briefly, 900 μl of ice-cold chloroform-methanol (1:2; volume/ volume (v/v)) was added to the plasma samples and vigorously vortexed for 1 min followed by shaking at 4 °C at 1200 RPM for 30 min using a thermomixer (Eppendorf, Hamburg, Germany). Equal amounts of ice-cold chloroform (300 μl) and ice-cold Milli-Q water (300 μl) were then added to break the phases. The samples underwent 2 min of vigorous vortexing and centrifugation at 12,000 RPM for 3 min. The lower organic phase, which contained the majority of lipids, was transferred to a fresh tube and kept on ice. The aqueous phase was re-extracted with 500 μl of ice-cold chloroform, vortexed again for 2 min and centrifuged at 12,000 RPM for 2 min. The organic fraction was collected again and combined with the first extraction. Pooled samples were then dried under vacuum using a miVac Duo Concentrator (Genevac Ltd, Ipswich, UK). Samples were stored at -80 °C until further analysis by MS.

### Lipid analysis by mass spectrometry

Dried lipid extracts were re-suspended in 200 μl of chloroform-methanol (1:1; v/v). Resuspended lipid extract were mixed 1:1 with internal standard mixture that was prepared separately and contained representative standards for each lipid class purchased commercially (Avanti Polar Lipids, Alabaster, AL; Echelon Biosciences, Salt Lake City, UT). The internal standard mixture consisted of the following lipid species: PA (1,2-dimyristoyl-glycero-3-phosphate or DMPA; final concentration 0.1 μg/ml), PE (1,2-dimyristoyl-glycero-3-phosphoethanolamine or DMPE; final concentration 4 μg/ml), PG (1,2-dimyristoyl-glycero-3-phospho-(1’rac-glycerol)) or DMPG; final concentration 0.1 μg/ml), PI (2-dioctanoyl-glycero-3-phosphoinositol or C8PI; final concentration 1 μg/ml), PS (dimyristoyl-glycero-phosphoserine or DMPS; final concentration 0.1 μg/ml), PC (1,2-dimyristoyl-glycero-3-phosphocholine or DMPC; final concentration 20 μg/ml), SM (lauroyl sphingomyelin or LSM; final concentration 12 μg/ml), Cer (N-heptadecanoyl-D-erythro-sphingosine or C17Cer; final concentration 0.5 μg/ml) and GluCer (D-glucosyl-β1-1’-N-octanoyl-D-erythro-sphingosine or C8GluCer; final concentration 0.5 μg/ml).

Sample (10 μl) was injected using an Agilent 1100 Autosampler system (Agilent Technologies, Santa Clara, CA) coupled to a triple quadrupole instrument ABI 4000 (Applied Biosystems, Foster City, CA) for MS analysis via shotgun approach. Lipids were ionized by ESI and each individual lipid molecular species was analyzed using a targeted multiple reaction monitoring (MRM) approach measuring over 100 unique transitions with known precursor/product mass-to-charge ratio (m1/m3)^26^. MRM is a widely used approach in the field of lipidomics as seen from previous studies^27^,^28^. The samples were first analyzed in the negative ion mode measuring transitions corresponding to PA, PE, PG, PI, PS and ganglioside mannoside 3 (GM3) and, afterwards, specific transitions for PC, SM, Cer and GluCer were analyzed in the positive ion mode. Chloroform-methanol (1:1; v/v), with 2% solution of 300 mM piperidine, was used as the mobile phase for lipids ionized in the negative ion mode and chloroform-methanol (1:1; v/v) was used for lipids ionized in the positive ion mode. Flow rate was maintained at 0.25ml/min. Instrumentation parameters for ionization were previously established^26^. The resolution of the instrument is of about 1,000 at *m/z* 760 and a mass accuracy of >200ppm. Run time was fixed at 2 min for each ion mode with continuum mode of data collection. The dwell time used was 20 ms for negative ion mode and 18 ms for positive ion mode. MRM method was used where Q1 and Q3 were set to pass molecularly distinctive precursor and product ions, whereby collisions were induces in Q2 using N_2_. All samples in each batch were analyzed in negative ion mode before switching polarity for positive ion mode analysis.

A complete list of the MRMs measured in this study and raw data for both pilot and validation studies are available in Table S1 in the Supplementary Online Material.

### Statistical analysis

SCIEX Analyst^®^ software was used to visualize and extract QTRAP Platform mass spectrometry raw data in its vendor format (.wiff), but its GUI lacks the feature for batch data extraction into text format. Analyst^®^ software provides a Microsoft Component Object Model (COM) interface for other clients, such as python to communicate with its components, to perform functions such as reading data values from its vendor format file. For this purpose, a simple python (release 2.5) script was written to extract ion intensities of every measured lipid species (for which the identity of each precursor/product mass-to-charge ratio (m1/m3) is already known), within a specified time range in a set of raw data files. Each lipid species is identifiable by its unique m1/m3 which we have measured using MRM mode, as described earlier. This script enabled the raw data in .wiff format to be converted into a tab-delimited text output file which contained samples in columns and ion intensities in rows. As such, average intensities (peak areas from 3s to 120s, according to visual inspection) of each glycerophospholipid and sphingolipid species were extracted using this python script. This script is available in Table S2 in the Supplementary Online Material.

Signal intensities were normalized to the representative spiked internal standards to obtain relative measurements, as previously described^29^. GM3 levels were normalized to the PI internal standard. For STL species, relative lipid concentrations were calculated by normalization to representative spiked internal standards.

Here, technical coefficient of variation (CoV) is defined as the variation due to the combined pre-processing of samples (process CoV) and analytical variation (instrument CoV). These values were calculated from normalized data (to spiked internal standard) from the validation study (n = 8, technical duplicates) as the ratio of standard deviation to average intensity, multiplied by 100^30^; a CoV cutoff of 20% was used before subsequent interpretation of any differences in lipid species.

A Friedman rank sum test^31^ was performed on each lipid species (with CoV less than 20%) for differences across the three time-points for both discovery pilot and validation studies independently, followed by analyses on the combined data set from both studies. For validation study samples, the mean values of duplicate measurements were used. Since rankings were computed within each subject among the three time-points, and samples from the same individuals were processed in the same batch, it was not necessary to perform any batch difference correction, even though data from multiple batches were combined for the analysis. To adjust for multiple testing while acknowledging the presence of correlation between lipid species, statistical significance is set at 0.01 for the discovery and validation panels, while requiring the combined evidence from both discovery and validation to be more significant than the naïve Bonferroni-corrected threshold of P < 3.9 × 10^−4^ at a traditional significance level of 0.05, given 128 lipid species were measured. Pearson correlation coefficients were calculated for the eight significant lipid species and the correlation plots were generated using R package “corrplot”. All analyses were performed using R (3.1) on a 64-bit Linux system^32^.

## Additional Information

**How to cite this article**: Begum, H. *et al.* Discovering and validating between-subject variations in plasma lipids in healthy subjects. *Sci. Rep.* **6**, 19139; doi: 10.1038/srep19139 (2016).

## Supplementary Material

Supplementary Figure

Supplementary Table S1

## Figures and Tables

**Table 1:** Table showing the –log10 p-values of the nine lipid species significantly altered across three timepoints t1 to t3 (unadjusted).

Lipid species	Discovery Pilot	Validation	Combined
PE (744)	4.63E-03	1.23E-04	6.97E-07
PE (742)	1.50E-03	5.85E-04	8.82E-07
PE (740)	8.05E-04	1.78E-03	1.50E-06
PE (716)	8.05E-04	4.32E-03	4.07E-06
PE (714)	8.05E-04	1.23E-04	1.06E-07
LPE (478)	3.35E-04	3.00E-04	1.06E-07
LPE (476)	3.35E-04	3.00E-04	1.06E-07
LPE (452)	9.80E-03	**3.19E-02**	6.80E-04
LPC (520)	9.80E-03	5.85E-04	7.33E-06

Nine lipid species are significantly altered across t1 to t3 across discovery pilot study, of which eight are replicated in the validation study (p < 0.01). The identities of these nine species are represented in the table across both independent and combined studies.
