# Supplementary material for: Discovering and validating between-subject variations in plasma lipids in healthy subjects
Source: Sci Rep. 2016 Jan 8;6:19139. doi: 10.1038/srep19139 (PMC4705481; doi:10.1038/srep19139)
Supplement: Supplementary Figure [file srep19139-s1.pdf]

Discovering and validating between-subject variations in plasma lipids in healthy subjects

Husna Begum[1,2,3,10], **Bowen Li**[4,10], **Guanghou Shui**[3,5], **Amaury Cazenave-Gassiot**[3], **Richie Soong**[6], **Rick Twee-Hee Ong**[4], **Peter Little**[3], **Yik-Ying Teo**[1,3,4,7,8,*], **Markus R Wenk**[1,2,3,9,*]

[1] NUS Graduate School for Integrative Science and Engineering, National University of Singapore, Singapore
[2] Department of Biochemistry, Yong Loo Lin School of Medicine, National University of Singapore, Singapore
[3] Life Sciences Institute, National University of Singapore, Singapore
[4] Saw Swee Hock School of Public Health, National University of Singapore, Singapore
[5] State Key Laboratory of Molecular Developmental Biology, Institute of Genetics and Developmental Biology, Chinese Academy of Sciences, Beijing, China
[6] Cancer Science Institute of Singapore, National University of Singapore, Singapore
[7] Genome Institute of Singapore, Agency for Science, Technology and Research, Singapore
[8] Department of Statistics and Applied Probability, National University of Singapore, Singapore
[9] Department of Biological Sciences, National University of Singapore, Singapore
[10] Both authors contributed equally to this work

Keywords: lipidomics, between-subject variation, Asian populations

* Co-corresponding author: Department of Biochemistry, Center for Life Sciences, 28 Medical Drive, National University of Singapore, Singapore 117546. Email: markus_wenk@nuhs.edu.sg. Tel: +65-6516 3624. Fax: +65 6777 3271.

Supplementary Table and Figure Legends

Supplementary Figure 1: Principal component analysis (PCA) plots of the normalized lipid data for both studies
(a) Pilot study subjects (eight subjects, three time-points each) and (b) Validation study subjects (nine subjects, three time-points each). Subjects that were able to be clearly differentiated from the rest of the cohort in the principle component plane (PC1, PC2) are indicated by the rectangular boxes across both studies. Data were centered but not scaled. Each subject is represented by a different symbol and each time-point measurement (t1 to t3) is represented by a different color, as indicated.

Table S1: Lipid data acquired in this study together with the list of lipid species both in positive and negative ion modes
MRM tab indicates list of all lipids measured in this study in both ion modes. Discovery and validation tabs indicate mass spectrometry lipid data acquired from both discovery pilot and validation studies.

Table S2: Python script used to extract ion intensities of every lipid species measured in this study
This script was written to allow data to be extracted from its raw .wiff format to a tab-delimited text output file which was used for any subsequent analysis.

Supplementary Tables and Figures

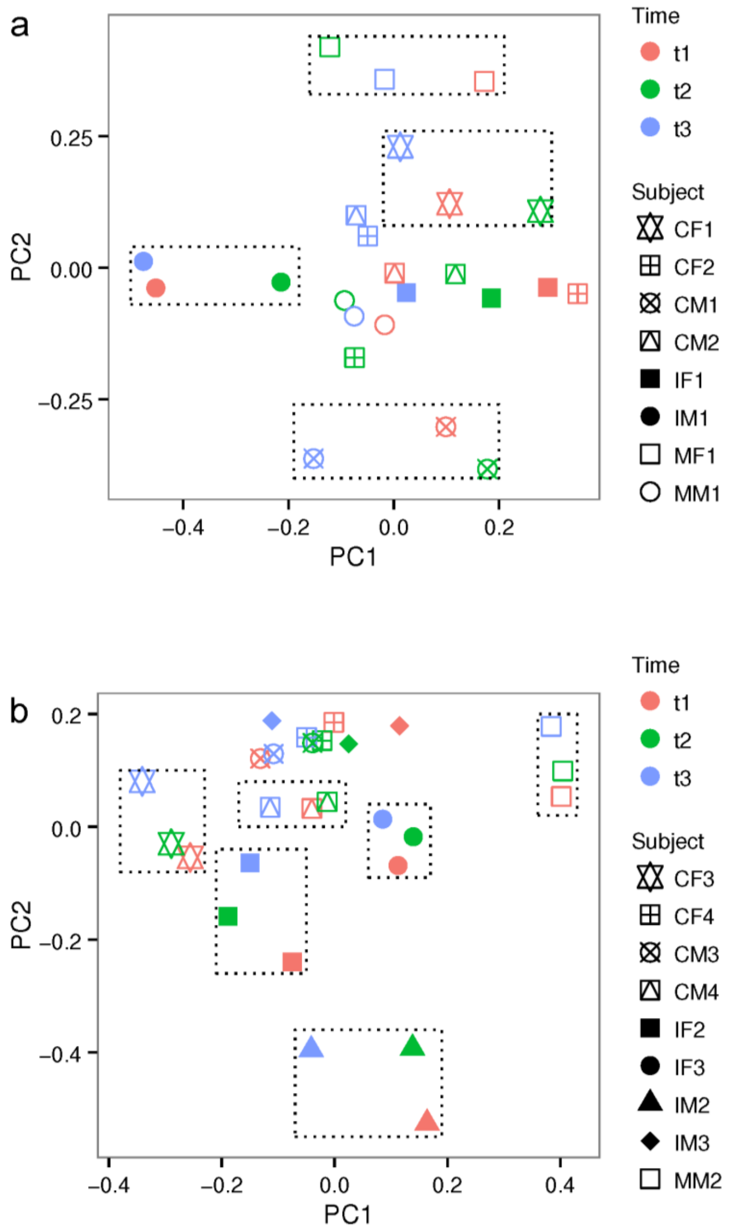

Supplementary Figure 1: Principal component analysis (PCA) plots of the normalized lipid data for both studies
(a) Pilot study subjects (eight subjects, three time-points each) and (b) Validation study subjects (nine subjects, three time-points each). Subjects that were able to be clearly differentiated from the rest of the cohort in the principle component plane (PC1, PC2) are indicated by the rectangular boxes across both studies. Data were centered but not scaled. Each subject is represented by a different symbol and each time-point measurement (t1 to t3) is represented by a different color, as indicated.

```
#!/usr/bin/env python

import sys,os,os.path
import re
import sha
import math
import tempfile
import csv
import string
from array import array
from base64 import b64encode
from urllib import quote

from optparse import OptionParser
import sys
import glob

if len(sys.argv) < 5:
	print '''Usage:
	MRM_all_mod.py numOfInput identifier start_time(s) end_time(s) filename > output_file
	Details:
	numOfInput -- the number of wiff files to extract, usually 1
	identifier -- usually 'notavailable'
	start_time, end_time -- time range, use end_time = 99999 to extract to the end
	filename -- wiff file name, must be full path, as in the example
	output_file -- can be full path or simply the file name

	Example:
```

```
	MRM_all_mod.py 1 notavailable 3.0 120.0  "C:\Documents And Settings\Admin\Desktop\test.wiff"
> "test.txt"

	'''

	sys.exit(1)


# input parameters

numInput=string.atoi(sys.argv[1])

identifier=sys.argv[2]

rtime_start=sys.argv[3]

rtime_end=sys.argv[4]

blank_identifier = 0


# identifier is used for selecting samples by pattern

if identifier == 'notavailable':

	blank_identifier = 1


p=re.compile(identifier,re.IGNORECASE)


sep=re.compile(":")


# m1/m3, samples names and intensity values

mass_list=[]

sample_list=[]

# use a composite key (sample name, m1/m3)

# reason: samples may be measured for different ion list

data={}

data_index_list=[]
```

```
# dispatch Microsoft Component Object Model interface
from win32com.client import Dispatch
theData = Dispatch('Analyst.FMANSpecData')
theTIC = Dispatch('Analyst.FMANChromData')

# process each input wiff file in a loop
for i in xrange(5,numInput+5):
    input_file=sys.argv[i]

    # initialize COM object
    theData.WiffFileName=input_file
    theTIC.WiffFileName=input_file

    # call COM functions
    theWF=theData.GetWiffFileObject()
    numSam=theWF.GetActualNumberOfSamples()

    # process each sample in a loop
    for j in xrange(1,numSam+1):
        # get sample name
        sampleName=theWF.GetSampleName(j)
        # for extracting all samples we append notavailble to file name
        # to match the search pattern
        if blank_identifier == 1:
            sampleName+=' notavailable'
        # match pattern in sample name
        m=p.search(sampleName)
```

```
if m: # pattern matched
    try:
        # in one period each m1/m3 will be scanned once
        numPeriod=theWF.GetActualNumberOfPeriods(j)
    except:
        continue

    # get the original sample name without pattern string
    end_pos=m.start()-1
    sample=sampleName[:end_pos]
    if sample_list.count(sample)==0:
        sample_list.append(sample)

    for k in xrange(0,numPeriod):
        numExperiment=theWF.GetNumberOfExperiments(j,k)
        for z in xrange(0,numExperiment):
            theTIC.SetToTIC(j,k,z)
            numTICs=theTIC.GetNumberOfDataPoints()

            if rtime_end==99999:
                endTime=theTIC.GetDataPointXValue(numTICs)*60
            else:
                endTime=rtime_end
            theData.SetSpectrum(j,k,z,rtime_start,endTime)
            numData=theData.GetNumberOfDataPoints()

            for z1 in xrange(1,numData+1):
                # parent ion mass
```

```
                        m1=theData.GetQ1Mass(z1)
                        # daughter ion
                        m3=theData.GetQ3Mass(z1)
                        # count
                        count=theData.GetDataPointYValue(z1)
                        mass='%2.1f/%2.1f' %(m1,m3,)
                        if mass_list.count(mass)==0:
                            mass_list.append(mass)
                        mass_index=mass_list.index(mass)
                        sample_index=sample_list.index(sample)
                        data[(sample_index,mass_index)]=count

# write header
outstr="\t"
for j in xrange(0,len(sample_list)):
    s=sample_list[j]
    outstr+=s
    outstr+="\t"
print outstr

# write table body
for i in xrange(0,len(mass_list)):
    m=mass_list[i]
    m_index=mass_list.index(m)
    outstr=m
    outstr+="\t"
    for j in xrange(0,len(sample_list)):
        s=sample_list[j]
```

```
        s_index=sample_list.index(s)
        if data.has_key((s_index,m_index)):
            outstr+="%2.4f" %(data[(s_index,m_index)],)
        else:
            outstr+="%2.4f" %(0,)
        outstr+="\t"
    print outstr
```

Table S2: Python script used to extract ion intensities of every lipid species measured in this study

This script was written to allow data to be extracted from its raw .wiff format to a tab-delimited text output file which was used for any subsequent analysis.
